# How Digital Technology Mediated the Effects of the COVID-19 Pandemic on Mental Health: The Good, the Bad, and the Indifferent

**DOI:** 10.3389/fdgth.2021.733151

**Published:** 2021-09-14

**Authors:** Lina Gega, Elias Aboujaoude

**Affiliations:** ^1^Department of Health Sciences & Hull York Medical School, University of York, York, United Kingdom; ^2^Department of Psychiatry & Behavioral Sciences, Stanford University School of Medicine, Stanford, CA, United States

**Keywords:** COVID-19, internet, internet gaming disorder, equity, suicide, depression, anxiety, telepsychiatry

## Introduction

For millions of people, the year of the COVID-19 pandemic represented an *annus horribilis*, marked by illness, loss, isolation, and unemployment. For those who managed to draw benefits from confinement and downtime, it was an *annus mirabilis*, providing the opportunity to spend more time with family, work creatively, take up hobbies, and reflect on priorities. For the rest, it was merely an *annus indifferentia* that saw them waiting with patience or boredom for time to pass, a vaccine to arrive, and conditions to improve. This divergence in experiences is reflected in the effects that the pandemic had on mental health: they were negative, positive, and neutral. Digital media—defined as information or content accessed and shared through a digital device or screen—have mediated some of the pandemic's effects and will continue to influence mental health—and mental health care—as we see ourselves out of the pandemic.

## Digital Media and Negative Effects of the Pandemic on Mental Health

Cross-sectional and longitudinal population surveys during the pandemic have suggested an increase in the frequency or severity of overall “distress,” occupational burnout among healthcare workers and caregivers, virus-focused as well as generalized anxiety, acute stress reactions, post-traumatic stress disorder, addiction, complicated grief, depression, and suicidality (1). This is attributed to many factors, including illness; excessive deaths; the loss of loved ones; limitations on movement (1); increased unemployment and worsening in finances (2); difficulties adjusting to remote work or education; disruptions in access to care; and increased abuse and violence within the home (3). We do not know the extent to which mental health has been affected by the overinflated use of digital media and difficulties with online learning/working during the pandemic, but problems already linked to digital technologies (4) may have worsened as screen time exploded, especially for those at risk, including adolescents with internet gaming disorder (5) and online gamblers with anxiety and depression (6). In addition, a new industry arose that took advantage of people's increased online accessibility to exploit their fears around the coronavirus through advertising fake cures and other scams (7).

Historical examples suggest that suicide rates increase during infectious outbreaks especially, for older generations during SARS in 2003 (8) and during the Great Influenza in 1918–1920 (9). On the contrary, during the COVID-19 pandemic, reports based on national and regional data from several countries indicate that overall suicide rates did not increase (10), but specific demographic and ethnic groups have been disproportionately affected. As a case in point, national data covering the entire Japanese population reported an increase in suicide rates by 37% among women and 49% for children and adolescents during the second wave of the pandemic (July to October 2020) (11). In a British study (12) that assessed mental health factors in a large population sample across three pandemic “waves,” younger adults (18–29 year olds) reported higher levels of suicidal ideation (12.5%) than those aged 30–59 years (8.4%; *p* = 0.002) and those ≥60 years (1.9%, *p* < 0.0001). In the same study, people from lower socioeconomic groups were also more likely to experience suicidal ideation (10.3 vs. 6.6%, *p* < 0.0001). Research from poorer parts of the world has raised concerns about an increase in suicide among people with existing mental health and addiction problems (13). Certain ethnic groups have also been disproportionately affected during the pandemic, as shown in a study of 1,079 suicide deaths between 2017 and 2020 in the US where mortality doubled among Black residents and was halved among White residents (14).

In the face of a pandemic that shifted much of life online, some negative effects on mental health, including suicidality, are likely to have been mediated by technology-specific factors. Selective or inappropriate media reporting of suicide (15)—especially celebrity suicide—has been linked to subsequent rises in suicidal behavior in the general population. How heavy online coverage of pandemic-related suicides may have spurred further suicidal behavior deserves to be explored. The digital divide may have also contributed to the differential effect of the pandemic on suicide, because many of the high-risk groups are also more digitally disadvantaged (16) and less likely to benefit from the protective mitigation of technology via remote work, recreation, social connection, and access to health services. The two extremes of digital media's invasive reach for some people vs. limited access for others may have amplified—or at least have not helped—suicidality and other mental health problems during the pandemic for specific population groups.

## Digital Media and Positive Effects of the Pandemic on Mental Health

Some people have reported positive experiences with the pandemic. In a longitudinal Australian survey of 1,370 participants (17), 70% reported experiencing at least one positive effect, most commonly citing the themes of “family time” (33%), “work flexibility” (29%), and “calmer life” (19%). Living with others (*p* = 0.045), being employed (*p* < 0.001), and female sex (*p* = 0.001) were associated with experiencing positive effects. Knowledge workers have been more privileged than manual, front-line and healthcare workers in experiencing beneficial effects. According to one survey (18), knowledge workers during the lockdown did 50% more activities through personal choice rather than because someone else asked them to; viewed their work as more worthwhile; and reported a decrease from 27 to 12% in the number of “tiresome” work tasks. Engaging in enjoyable, purposeful, and rewarding activities, and increasing social connectedness, are not only protective factors for mental health, but also an established mechanism for improving depression, known as behavioral activation (19).

Another “silver lining” of the pandemic when it comes to mental health is the growing use of telepsychiatry and digital interventions, whose clinical utility and cost-effectiveness have been shown in comparison to “no treatment” and in some cases through metanalytic comparisons with the in-person “gold standard” treatment (20–22). Digital media can offer a sustainable solution to the chronic problem of limited access to mental health care, by helping services overcome geographical barriers and make the most out of the available workforce through remote consultations, task-shifting, and supported self-management. These offerings had long been limited by poor adoption rates, high attrition, forbidding medico-legal risks, resistance by insurers and other payers, and little formal support and guidance from professional organizations (23). But the disruption wrought by the pandemic meant that many patients and providers who would have not considered using technology otherwise had an opportunity to try it, often with encouraging results, even if the benefit was not evenly distributed across social and socioeconomic groups due to differences in digital access and skills (24). The pandemic is prompting important work around tele-mental-health interventions in routine care for common conditions like anxiety and mild-moderate depression, but it also opens the door to digitally enabled care for more challenging conditions such as psychotic disorders and suicidal depression (25). This work will continue post-pandemic as more data is collected and analyzed and as the benefits as well as the drawbacks of the transition to remote delivery of mental health care become clearer.

## Digital Media and Neutral Effects of the Pandemic on Mental Health

Fortunately, the majority of people have not developed mental health conditions during the pandemic (2) and are unlikely to do so in its immediate aftermath (26), casting doubt on predictions and assertions of a “mental health pandemic.” This does not negate the increase in individual pain and the massive number of people whose mental health is, and will be, affected. As a historical case in point, the Great War of 1914–1918 did not appear to result in an increase in population-level suicide rates (9), but post-traumatic stress disorder was a major health and social issue for war veterans.

Does a “neutral effect” make psychological sense? The pandemic has changed the way we view ourselves, the world, and the future; a concept known as “cognitive triad” (27). On the one hand, viewing ourselves as more vulnerable, the world as more dangerous, and the future as more uncertain in response to the pandemic, can fuel mental health problems. On the other, viewing ourselves as more resilient, the world as a global community that has pulled together, and the future as a better place where we will not take everything for granted, can reinforce mental well-being. The combination of vulnerability and resilience, threat and safety, uncertainty and hope, may produce a neutral “net effect” on mental health, as different approaches to the pandemic “average out” between different people in a population, and for the same person at different times.

The ability to share experiences and connect with others during periods of a crisis can have a “neutralizing” influence on psychological distress. Digital media, from social networks to massively multiplayer online games (MMOGs), have afforded us the ability to “share and connect”, thereby potentially buffering some negative pandemic effects and resulting in an overall neutral impact on mental health. A Canadian study with adolescents found that feeling socially connected during lockdown protected them against poor mental health (28). Another example from a national UK survey—albeit an observation rather than proof of mediation—was that adolescents experienced less depression and anxiety symptoms and at the same time reported more digitally enabled social contact than primary school children (29).

Even if the overall impact of the pandemic on mental health proves to be neutral for most people, or as an average across the whole population, this does not generalize to all the population's constituent groups—defined by gender, ethnicity, age, occupation, or pre-existing health and social needs. The negative effects and risks of the pandemic on mental health are more pronounced among ethnic minorities, women (especially those living with children), younger adults, people with pre-existing mental health or addiction problems, people in lower income households, front-line workers, and, of course, those who fell seriously ill or were left bereft (30). For these groups, we need to scale up the provision of mental health services, in which digital interventions can play a big part. Unfortunately, the caveat is that many of these high-risk groups are also digitally disadvantaged (16).

## Discussion

Digital media have mediated both positive and negative effects of the COVID-19 pandemic on mental health. Surveys capturing these effects are not infallible, since they are based on self-report questionnaires that are subject to misinterpretation, recall bias, and socially influenced answers. Non-probability and convenience sampling methods do not adequately capture the effects of the pandemic on the most vulnerable population groups. In the absence of perfect data, making best use of what is currently available can guide our decisions and inform our plans for mental health care; however, further surveys need to focus on population groups for whom we now know that the pandemic is likely to have negative, pronounced, and long-term effects.

The legacy of the pandemic on mental health may not be as catastrophic as it has been feared, and population-level mental health outcomes may end up no different post- than pre-pandemic. An increase in psychological distress and psychopathology as a direct reaction to a stressful context can be expected to subside as the stressor recedes (31), with only a fraction of cases evolving into diagnosable, long-lasting conditions (32). Still, even small but persistent increases in the frequency or severity of psychiatric symptoms, when considered across the billions of individuals touched by the pandemic, can add significantly to the global burden of mental illness. This strengthens the argument for investing in mental health services around the world, especially because they were already so limited, as illustrated by the fact that only 43.8% of US adults with mental illness received treatment pre-pandemic (33). Also, we should not be complacent about the pandemic's adverse, disproportionate, and long-term effects on specific groups of people. The pandemic is a major risk factor, which will magnify existing health disparities and increase the global burden of mental illness.

The world will undoubtedly be planning for the next pandemic by putting in place robust systems to identify infectious diseases, prevent their spread, and treat their casualties. By the same token, even if the spike in psychopathology eases after the pandemic, we need to invest in mental health systems that recognize and respond effectively to increased risk and symptoms and that treat people with existing vulnerabilities. As Gilbody and colleagues noted in their compelling commentary (34), we have been successful at describing the nature of the impact of COVID-19 on mental health, but less successful at generating and evaluating solutions to mitigate these impacts. The mammoth scale of the COVID-19 vaccination can only be mirrored in mental health with the use of digitally enabled services and interventions. The pandemic paved the way for digital technologies to become an acceptable and sustainable way of working, learning, caring, and interacting with each other; we must capitalize on the momentum we have gained about these technologies during this pandemic to scale up provision of mental health care in its aftermath.

## Conclusion

Over the last year and a half, people have reacted with feelings of sadness, relief, indifference, or a complex mix thereof as the world carried out a natural experiment in mass and rapid deployment of technology for work, education, treatment, social interaction, and seemingly everything in between. What this experiment has meant for mental health, and what it portends for the future, is only starting to crystalize and will become clearer as more data are analyzed and experiences are processed. While many mental health effects have been negative and pronounced for specific population groups, we can draw comfort from the neutralizing and positive effects that technology has in some cases mediated. The pandemic has been an accelerator for digital technologies; we should capitalize on their benefits, while mitigating their risks and associated inequalities, in order to reach those people who need mental health care the most.

## Author Contributions

LG conceived the outline of this paper and drafted the first version. EA reviewed and revised a second draft of the paper. Both authors reviewed and revised subsequent drafts of the manuscript and created the final version.

## Conflict of Interest

LG received grants from the National Institute for Health Research, the UK Research and Innovation, the Medical Research Council and the Wellcome Trust to lead research relating to digital technologies and mental health. LG leads the digital mental health theme at the Mental Health and Addiction Research Group, University of York. EA reports royalties from Oxford University Press, Cambridge University Press, University of California Press and W.W. Norton for work relevant to digital mental health. The views expressed are the authors' own.

## Publisher's Note

All claims expressed in this article are solely those of the authors and do not necessarily represent those of their affiliated organizations, or those of the publisher, the editors and the reviewers. Any product that may be evaluated in this article, or claim that may be made by its manufacturer, is not guaranteed or endorsed by the publisher.
